# Effect of *Latilactobacillus curvatus* HY7601 and *Lactiplantibacillus plantarum* KY1032 on Serum Triglyceride Levels and the Gut–Metabolic Axis: A Randomized, Double-Blind, Placebo-Controlled Clinical Trial

**DOI:** 10.3390/nu18111713

**Published:** 2026-05-27

**Authors:** Eun-Ji Kim, Dong-Ki Hong, Il-Dong Choi, Jae-Jung Shim, Jae-Hwan Lee, Woo-Kil Jung

**Affiliations:** 1 R&BD Center, hy Co., Ltd., 22, Giheungdanji-ro 24beon-gil, Giheung-gu, Yongin-si 17086, Republic of Korea; ejkim@hy.co.kr (E.-J.K.); dkhong@hy.co.kr (D.-K.H.); cid1010@hy.co.kr (I.-D.C.); jjshim@hy.co.kr (J.-J.S.); 2 Department of Family Medicine, Vievis Namuh Hospital, 627, Nonhyeon-ro, Gangnam-gu, Seoul 06117, Republic of Korea

**Keywords:** *Latilactobacillus curvatus*, *Lactiplantibacillus plantarum*, triglyceride, lipid profile, probiotics, metabolic health, gut–metabolic axis, network analysis

## Abstract

**Background/Objectives:** Hypertriglyceridemia is a critical cardiovascular risk factor, and the probiotic combination of *Latilactobacillus curvatus* HY7601 and *Lactiplantibacillus plantarum* KY1032 (HY+KY) has emerged as a potential therapeutic strategy, though clinical validation in adults with mild hypertriglyceridemia (HTG) is needed. **Methods:** In this randomized, double-blind, placebo-controlled, 12-week trial, a total of 100 overweight participants with mild HTG were randomized (*n* = 50 per group). Ultimately, 80 participants completed the study without major protocol violations and were evaluated in the Per-Protocol Set (probiotics group: *n* = 41; placebo group: *n* = 39). Primary outcomes included changes in serum lipid profiles such as triglycerides (TG) and LDL cholesterol (LDL), metabolic biomarkers, and gut microbiota composition analyzed via 16S rRNA gene sequencing. **Results:** HY+KY supplementation led to significant reductions in serum TG (158.61 ± 23.17 to 139.54 ± 54.31 mg/dL, *p* = 0.009) and LDL (129.22 ± 28.45 to 111.34 ± 21.03 mg/dL, *p* = 0.005) compared to baseline, while the placebo group showed no significant changes. Furthermore, the HY+KY group exhibited a significant increase in Apolipoprotein CII (ApoC2, *p* = 0.034) and a reduction in fasting glucose levels (*p* = 0.021). Microbiome analysis revealed that HY+KY significantly increased alpha diversity (Shannon index, *p* = 0.012) and significantly altered the microbial community structure (beta diversity, *p* = 0.015). Co-occurrence network analysis identified *Lactiplantibacillus* as a highly connected central node that is strongly associated with the favorable shifts in clinical biomarkers. **Conclusions**: HY+KY supplementation was associated with improved fasting TG and LDL profiles in adults with mild HTG, alongside coordinated changes in ApoC2, fasting glucose, and gut microbiota structure. These findings support the potential of probiotic supplementation as a preventive nutritional approach in borderline HTG.

## 1. Introduction

Cardiovascular disease (CVD) remains the leading cause of global morbidity and mortality, posing a significant concern for healthcare systems worldwide [1]. Dyslipidemia, specifically hypertriglyceridemia (HTG), is an important risk factor that independently drives atherosclerotic progression [2,3,4]. Elevated serum triglycerides (TG) directly contribute to the accumulation of atherogenic remnant lipoproteins and the promotion of systemic inflammation [5,6]. Despite its clinical significance, the global prevalence of HTG is steadily increasing, largely driven by modern dietary patterns and sedentary lifestyles [6]. While pharmacological treatments such as statin are available, potential side effects and long-term adherence challenges underscore the urgent need for effective non-pharmacological adjuncts, such as functional food-based interventions, to restore lipid homeostasis [7,8,9].

The gut microbiota has emerged as an important regulator of host energy homeostasis and lipid metabolism [10,11,12]. Recent evidence has demonstrated that gut dysbiosis is closely associated with the pathogenesis of various metabolic disorders, including hyperlipidemia and obesity [13,14]. These interactions are primarily mediated by microbial-derived products such as metabolites, which modulate host metabolism by inducing mild immune responses [15]. In particular, short-chain fatty acids (SCFAs) function as key microbial-derived signaling molecules that regulate lipid synthesis and fatty acid oxidation via host metabolic pathways, including G-protein-coupled receptor-mediated mechanisms [16,17]. Consequently, change in the gut microbial environment has emerged as a potential therapeutic strategy for restoring systemic lipid homeostasis and alleviating metabolic dysfunction [10,18].

Research on the diverse functional properties of probiotics has expanded significantly in recent years [19,20]. In particular, probiotics have demonstrated substantial potential for modulating lipid profiles by lowering serum TG and cholesterol levels [12,21]. Multiple clinical studies have established the efficacy of specific *Lactobacillus* and *Bifidobacterium* species in the management of dyslipidemia [22,23]. However, it is crucial to evaluate whether the mechanisms observed in controlled in vitro settings translate effectively into the high-dimensional and competitive microbial ecosystem of the human gut [24]. Unlike isolated experiments, the intestinal environment involves intricate cross-feeding and antagonistic interactions between the administered strains and the indigenous microbiota [25,26]. Therefore, employing analytical tools like correlation heatmaps and network analysis is crucial to visualize the dynamic interplay within the gut–metabolic axis [27,28,29]. These topological frameworks move beyond simple abundance comparisons to offer a holistic understanding of how probiotics reconfigure the microbial ecosystem to improve systemic lipid metabolism [30,31].

*L. curvatus* HY7601 has been characterized for its ability to promote fatty acid oxidation and enhance lipid catabolism, thereby reducing cellular lipid accumulation [32]. Independently, *L. plantarum* KY1032 has demonstrated potent anti-adipogenic and cholesterol-lowering activities [33]. In addition, HY+KY supplementation has been shown to decrease serum TG levels by downregulating hepatic lipogenic markers such as SREBP-1c and FAS, while enhancing pathways related to fatty acid oxidation [34]. These metabolic benefits are accompanied by favorable alterations in gut microbiota composition, suggesting a mechanistic link between microbial modulation and lipid metabolism [32]. Based on the complementary roles of each strain, we hypothesized that their combination would exert a synergistic effect on lipid homeostasis.

While these previous studies on the HY+KY primarily focused on body fat reduction or the management of established HTG, its impact on the earlier stages of lipid imbalance has not been fully explored. To address this gap, we conducted a 12-week randomized, double-blind, placebo-controlled trial to evaluate HY+KY efficacy in subjects with mild HTG. Also, this study investigated the intricate relationship between gut microbiome alteration and lipid metabolism markers within the intestinal environment. Ultimately, our results may provide a scientific basis for the development of HY+KY as a functional ingredient to support healthy lipid management through the modulation of the gut microbiome.

## 2. Materials and Methods

### 2.1. Study Participants

To evaluate the efficacy of the probiotics mixture of HY+KY in improving blood TG levels, adult men and women aged 19 to under 70 were enrolled if they met the following criteria: (1) fasting blood TG levels between 150 and 199 mg/dL; (2) fasting blood TG levels between 120 and 149 mg/dL accompanied by total cholesterol (TC) levels between 200 and 239 mg/dL. Participants were recruited from Vievis Namuh Hospital (Seoul, Republic of Korea), and all participants provided written informed consent forms.

Exclusion criteria included the following: current treatment for severe systemic diseases (cardiovascular, immune, respiratory, gastrointestinal, hepatobiliary, urinary, neurological, musculoskeletal, psychiatric, infectious, or malignant); body mass index (BMI) < 20 kg/m^2^ or >35 kg/m^2^; use of medications or supplements affecting hormonal or lipid metabolism, or intake of antibiotics, intestinal regulators, or probiotics within 3 months prior to screening; diagnosis of hypercholesterolemia or a history of drug or substance abuse; history of gastrointestinal surgery; hypertension, defined as systolic blood pressure ≥ 140 mmHg or diastolic blood pressure ≥ 90 mmHg; glycated hemoglobin (HbA1c) ≥ 6.5% or diagnosis of diabetes mellitus; abnormal thyroid function; estimated glomerular filtration rate (eGFR) < 60 mL/min/1.73 m^2^; aspartate aminotransferase (AST) or alanine aminotransferase (ALT) levels exceeding three times the upper limit of normal; excessive smoking, as judged by the investigator; pregnancy or lactation; participation in any other human clinical trial within three months prior to screening.

Participants were instructed to maintain their usual levels of physical activity and dietary intake throughout the study period. Physical activity was assessed using the Global Physical Activity Questionnaire (GPAQ). Specifically, individuals whose activity levels shifted between the ‘low’ and ‘high’ categories (either from low to high or high to low) from baseline to week 12 were removed. And dietary intake was monitored using dietary records and analyzed with the Computer-Aided Nutritional Analysis Program (CAN-pro; Korean Nutrition Society, Seoul, Republic of Korea).

### 2.2. Study Design

This study was a randomized, double-blind, placebo-controlled clinical trial conducted to evaluate the effect of a 12-week supplementation with a probiotic mixture of HY+KY on blood TG levels. This study was approved by the Institutional Review Board (IRB) of Vievis Namuh Hospital (IRB No. VNIRB-202412) and conducted in accordance with the principles outlined in the Declaration of Helsinki.

The sample size was determined by a priori power analysis. To achieve 80% power at a 5% significance level, a minimum of 37 participants per group was required based on a conservative estimate of the expected difference in TG levels. Considering a potential dropout rate of 25%, a total of 100 participants (50 per group) were enrolled to ensure sufficient statistical power [35]. A total of 100 participants who met the inclusion and exclusion criteria during the screening visit were randomized in a 1:1 ratio to the HY+KY (*n* = 50) or placebo (*n* = 50) group according to a randomization schedule generated by an independent external statistician using the SAS system (version 9.4; SAS Institute, Cary, NC, USA) at the Visit 2. To ensure allocation concealment, the statistician prepared sequentially numbered, opaque, sealed envelopes. After the clinical investigators in hospital completed the enrollment and confirmed eligibility, participants were assigned to their respective groups by opening the envelopes in numerical order. The allocation remained double-blinded to both the investigators and participants; the randomization codes were maintained securely and were only revealed during the blind-breaking meeting following the completion of the trial and the final data lock.

**Figure 1:**
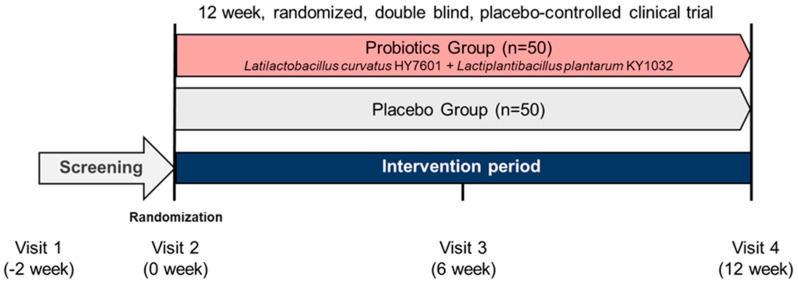
Study design and flow chart of the clinical trial. This was a 12-week, randomized, double-blind, placebo-controlled clinical trial designed to evaluate the effects of a probiotic mixture (*L. curvatus* HY7601 + *L. plantarum* KY1032) on blood TG levels.

Participants attended four study visits over a 12-week period at 6-week intervals, including the screening visit: Visit 1 (screening, week 2), Visit 2 (week 0), Visit 3 (week 6), and Visit 4 (week 12). An overview of the study design is represented in Figure 1.

### 2.3. Study Products

The HY+KY strains isolated from kimchi were provided by hy Co., Ltd. (Yongin-si, Republic of Korea). Participants in the HY+KY group received two capsules daily, each containing 500 mg, for a total daily dose of 1 × 10^10^ CFU of probiotics. The HY+KY capsules contained lactic acid bacteria, as well as excipients necessary for capsule production, including magnesium stearate and microcrystalline cellulose.

Participants in the placebo group received two capsules daily, each containing 500 mg. These placebo capsules were identical in appearance and color to the HY+KY capsules, but contained lactose in place of probiotics, with the same excipient composition. Participants in the study received a 6-week supply of the products at Visit 2 and Visit 3. Probiotic viability was strictly monitored at three-month intervals throughout the study period. Quality control assessments confirmed that the target dosage of 1 × 10^10^ CFU was consistently maintained until the final participant completed the 12-week intake period. Any remaining products were returned at the subsequent visit for adherence assessment.

### 2.4. Safety

To evaluate the safety of the 12-week intervention, all participants underwent comprehensive monitoring at baseline and at the end of the study. At the beginning of the study, demographic information (sex, age, alcohol consumption, and smoking status), vital signs (blood pressure and heart rate), and anthropometric measurements were collected for all subjects. To verify the safety of the investigational product, clinical laboratory tests—including electrocardiography (ECG), hematology, blood chemistry, and urinalysis profiles—were performed at baseline and at the end of the study to examine changes before and after the intervention period. Furthermore, participants were assessed for the occurrence of adverse events (AEs) at each visit.

### 2.5. Efficacy Outcomes

Efficacy assessments were performed through medical examinations at Vievis Namuh Hospital (Seoul, Republic of Korea). The lipid profile measured during these examinations included fasting serum TG, TC, low-density lipoprotein cholesterol (LDL), and high-density lipoprotein cholesterol (HDL), all of which were quantified using colorimetric methods with appropriate commercial kits. Additionally, parameters associated with TG synthesis and metabolic change, such as apolipoprotein B (ApoB), apolipoprotein CII (ApoC2), serum glucose (Glu), and free fatty acids (FFA), were analyzed using Hitachi 7600 automatic analyzer (Hitachi High-Tech Corporation, Tokyo, Japan). Serum insulin (Ins) concentrations were determined via enzyme-linked immunosorbent assay (ELISA) kit (Thermo Fisher Scientific, Waltham, MA, USA).

The timing of these measurements varied across study visits. Fasting TG and TC levels were determined at Visit 1, Visit 3, and Visit 4. LDL and HDL levels were measured at Visits 2, 3, and 4, while the concentrations of ApoB, ApoC2, Glu, Ins, and FFA were assessed at Visits 2 and 4. To ensure the reliability of the clinical data, participants were required to adhere to a strict pre-test protocol. This included abstaining from alcohol consumption, high-fat meals, and strenuous physical exercise for 24 h prior to each scheduled visit. All blood samples were collected following a minimum of a 12 h overnight fast.

### 2.6. Statistical Analysis

Efficacy analyses were conducted on the Per-Protocol Set (PPS), which included participants who completed the clinical trial without any major protocol violations that could influence the study outcomes. To ensure the robustness of the findings and address potential dropout biases, secondary supportive analyses were performed on the Full Analysis Set (FAS) using identical statistical model. Continuous variables were presented as mean ± standard deviation (SD), while categorical variables were expressed as numbers and percentages.

For efficacy analyses, within-group comparisons between baseline and follow-up visits (week 6 and week 12) were performed using the paired *t*-test for normally distributed data or the Wilcoxon signed-rank test for non-normally distributed data. To evaluate the intergroup differences in the changes in lipid profiles, a linear mixed-effects model (LMM) was performed. The model included fixed effects for treatment group, time, and their interaction (treatment-by-time), with subject included as a random effect to model within-subject dependencies. Furthermore, age, sex, BMI, dietary and physical activity (PA) were incorporated as covariates in the model to adjust for potential confounding effects.

Statistical analyses for safety data were conducted to compare changes between the test and control groups. For continuous variables, the two-sample *t*-test or the Wilcoxon rank-sum test was employed, depending on the normality of the data distribution. Categorical data were evaluated using the Chi-square test or Fisher’s exact test to assess independence. Urinalysis data were analyzed using the McNemar test to assess significant shifts within each group. All statistical analyses were performed using SAS^®^ software (version 9.4; SAS Institute, Cary, NC, USA).

### 2.7. Fecal Microbiome Analysis

#### 2.7.1. Fecal Sample Collection and Preparation

After obtaining written informed consent for fecal collection, stool samples were collected twice during the study period. Participants who passed the screening at Visit 1 collected their fecal samples prior to the initiation of the test product intake and submitted them at Visit 2. Subsequently, the second collection was performed at Visit 4, following the completion of the 12-week clinical trial. All stool samples were submitted in ice bags to maintain temperature and were immediately stored at −80 °C until analysis.

Genomic DNA was extracted using the MoBio PowerSoil DNA Isolation Kit (Qiagen, Hilden, Germany) in accordance with the manufacturer’s instructions. The extracted DNA was quantified using a Victor Nivo Multimode Microplate Reader (PerkinElmer, Waltham, MA, USA). The purity of the extracted DNA was verified using a NanoPhotometer N120 system (Implen GmbH, Munich, Germany) by measuring the A260/280 and A260/230 ratios, ensuring that the samples were of sufficient quality for microbiome analysis.

#### 2.7.2. 16S rRNA Amplicon Sequencing

To analyze the microbiome profile, the V3–V4 variable regions of the bacterial 16S rRNA gene were amplified and sequenced using the Illumina MiSeq platform (Illumina Inc, San Diego, CA, USA). Genomic DNA (gDNA) was amplified by polymerase chain reaction (PCR) using bacterial universal forward and reverse primers. The initial PCR was performed under the following thermal cycling conditions: an initial denaturation at 95 °C for 3 min; followed by 25 cycles of denaturation at 95 °C for 30 s, annealing at 55 °C for 30 s, and extension at 72 °C for 30 s; with a final extension at 72 °C for 5 min.

The universal primer set, which included Illumina adapter overhang sequences for the first amplification, consisted of V3-F (5′-TCGTCGGCAGCGTCAGATGTGTATAAGAGACAGCCTACGGGNGGCWGCAG-3′) and V4-R (5′-GTCTCGTGGGCTCGGAGATGTGTATAAGAGACAGGACTACHVGGGTATCTAATCC-3′). The resulting first-stage PCR products were purified using AMPure beads (Agencourt Bioscience, Beverly, MA, USA).

For the final library construction, 2 μL of the purified first-stage PCR product was used as a template for the second round of PCR. The thermal cycling conditions for the second stage were identical to those of the first stage, except that the number of cycles was reduced to 10. The finalized PCR products were then submitted to Macrogen (Seoul, Republic of Korea) for sequencing on the Illumina MiSeq platform.

#### 2.7.3. Analysis of Amplicon Sequence Variants (ASVs)

Sequencing data were analyzed using the QIIME 2 platform, incorporating chimera detection, quality filtering, and sequence denoising via the DADA2 pipeline [36]. Taxonomic classification was assigned to ASVs based on the SILVA SSU databases version 138.1. To account for variations in sequencing depth across samples, the raw ASV counts were normalized to relative abundance (%) by dividing the counts of each ASV by total number of reads per sample.

To evaluate changes in microbial community diversity within the placebo and HY+KY groups before and after intervention, alpha diversity was assessed using the Shannon and Simpson indices. Beta diversity was calculated based on the Bray–Curtis distance matrix to determine dissimilarities between samples, and the resulting shifts in microbial community structure were visualized through principal coordinate analysis (PCoA) plots. Statistical significance of the beta diversity between groups was evaluated using Permutational Multivariate Analysis of Variance (PERMANOVA). All statistical analyses and visualizations were performed using R studio software (version 4.3.3, https://posit.co/download/rstudio-desktop/ (accessed on 27 March 2026)), primarily utilizing the vegan and phyloseq packages.

### 2.8. Correlation Analysis Between Clinical Parameters and Gut Microbiota

#### 2.8.1. Heat Maps Analysis

To mitigate the risk of false positives from multiple comparisons and to focus on ecologically functional taxa, NGS data was filtered prior to correlation analyses. Pairwise Spearman’s rank correlation coefficients and their *p*-values were calculated between the top 25 genera that selected based on their mean relative abundance across all samples and clinical parameters using the Hmisc package in R studio. In heatmaps, the color gradient represents the correlation coefficient, with red indicating a positive correlation and blue indicating a negative correlation. To ensure statistical reliability, only correlations with a *p* < 0.05 were considered statistically significant and displayed with asterisk mark (*).

#### 2.8.2. Network Analysis

To further elucidate the complex interactions and identify hub nodes associated with clinical improvements, a co-occurrence network analysis was performed based on the Spearman correlation matrix. Network construction and topological analysis were conducted using the igraph, tidygraph, and ggraph in R studio. Nodes in the network represent bacterial genera or clinical variables, while edges represent significant correlations. To reduce noise and focus on robust interactions, only edges satisfying a significance threshold of *p* < 0.05 and a correlation strength threshold |r| > 0.3 were retained. To account for multiple hypothesis testing and reduce the risk of Type I errors, all *p*-values were adjusted using the Benjamini–Hochberg False Discovery Rate (FDR) procedure.

Network data were imported into Cytoscape software (version 3.10.3) for visualization and topological analysis. The network nodes were arranged using the Attribute Circle Layout. Furthermore, topological network parameters were calculated using the Analyze Network tool within Cytoscape to quantitatively identify hub nodes within the network.

## 3. Results

### 3.1. The Characteristics of Study Participants

**Figure 2:**
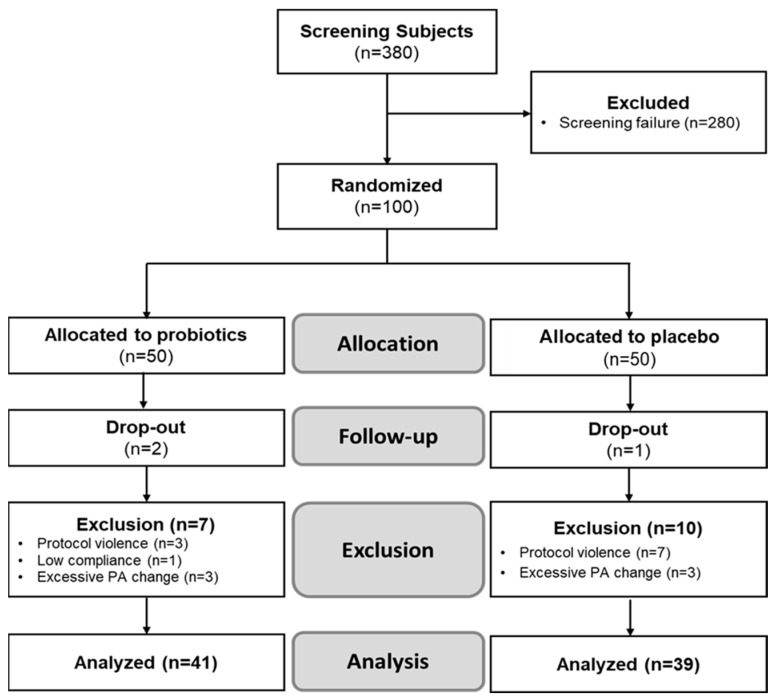
CONSORT flow diagram of the study participants. Flow of participants through each stage of the clinical trial, including screening, allocation, follow-up, exclusion and analysis. Participants who fulfilled all eligibility criteria without exclusion were included in the final analysis using the PPS.

A total of 380 subjects were initially screened, of whom 280 were excluded for failing to meet the eligibility criteria. Consequently, 100 participants were randomized into either the HY+KY group (*n* = 50) or the placebo group (*n* = 50) (Figure 2). During the intervention period, two subjects in the HY+KY group and one in the placebo group were excluded after withdrawing their written informed consent. In the HY+KY group, three participants were excluded due to protocol violations, one for low compliance, and three due to excessive changes in physical activity. As a result, 41 subjects in the HY+KY group were included in the final PPS analysis. In the placebo group, seven participants were excluded for protocol violations and three for excessive changes in physical activity, leaving 39 subjects for the PPS analysis. The baseline demographic characteristics of the participants are presented in Table 1, and no significant differences were observed between the HY+KY and placebo groups.

### 3.2. Safety Assesments

To evaluate the safety of placebo or probiotic consumption during the trial, safety indicators were measured before and after the intervention (Table 2). No statistically significant intergroup differences were observed in hematological, blood chemistry, or urinalysis variables between the placebo and HY+KY groups at either baseline or week 12 (*p* > 0.05). Furthermore, there were no significant differences in the magnitude of change between the groups during the intervention period (*p* > 0.05). No serious adverse events were reported for any participants who consumed the placebo or probiotics at least once (defined as the safety set), including those excluded due to protocol violations.

### 3.3. Changes in Serum Lipid Profiles

**Figure 3:**
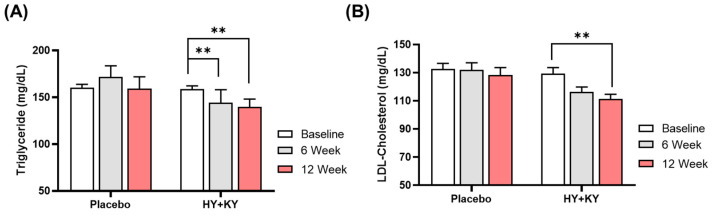
Results of serum lipid profiles during the 12-week intervention. The graphs show (**A**) serum TC and (**B**) LDL levels were measured at baseline, 6-week, and 12-week. Data are presented as mean ± SD. *p*-values were analyzed using the Wilcoxon signed-rank test. ** *p* < 0.01.

Figure 3 and Table S1 summarize the changes in serum lipid profiles over the 12-week intervention period. In the HY+KY group, serum TG levels significantly decreased from 158.61 ± 23.17 mg/dL at baseline to 139.54 *±* 54.31 mg/dL at 12 weeks (change: −19.07 *±* 58.46 mg/dL, *p* = 0.009). A significant reduction in TG was also observed at the 6-week mark (change: −14.39 *±* 84.27, *p* = 0.002). Furthermore, LDL levels in the HY+KY group also showed a significant reduction from 129.22 ± 28.45 mg/dL at baseline to 111.34 ± 21.03 mg/dL at 12 weeks (change: −17.88 ± 35.35, *p* = 0.005). In contrast, the placebo group showed no statistically significant changes in TG (*p* = 0.081), TC (*p* = 0.851), LDL (*p* = 0.669) and HDL (*p* = 0.862) after 6 weeks and 12 weeks. In the FAS analysis (*n* = 97), the significant reduction in TG levels (*p* = 0.022) was maintained in the HY+KY group, while LDL (*p* = 0.064) exhibited consistent trends toward improvement (Table S3).

### 3.4. Changes in Apolipoproteins and Metabolic Biomarkers

**Figure 4:**
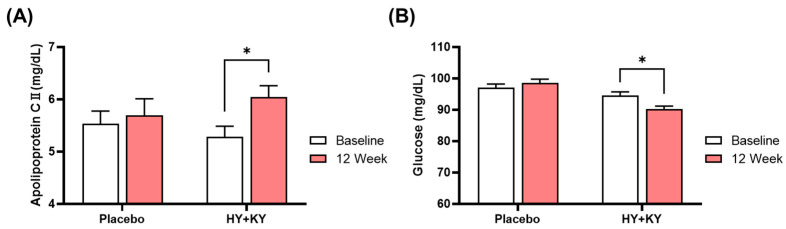
Results of serum ApoC2 and Glu levels after 12-week intervention. The graphs show (**A**) serum ApoC2 and (**B**) Glu levels were measured at baseline, and 12-week. Data are presented as mean ± SD. *p*-values were analyzed using the Wilcoxon signed-rank test. * *p* < 0.05.

Figure 4 and Table S2 summarize the changes in apolipoproteins and metabolic biomarkers after the 12-week intervention. In the HY+KY group, serum ApoC2 levels significantly increased from 5.28 ± 1.32 mg/dL at baseline to 6.04 ± 1.42 mg/dL at 12 weeks (change: 0.76 ± 1.66 mg/dL, *p* = 0.034). Furthermore, fasting Glu levels in the HY+KY group showed a significant reduction from 94.51 ± 7.92 mg/dL at baseline to 90.27 ± 5.96 mg/dL at 12 weeks (change: −4.24 ± 9.54 mg/dL, *p* = 0.021). In contrast, the placebo group exhibited no significant changes in any of the measured variables. In the FAS analysis (*n* = 97), the significant reduction in ApoC2 levels (*p* = 0.048) was maintained in the HY+KY group, while Glu (*p* = 0.294) showed reduction without statistical significance (Table S3).

### 3.5. Effects on Gut Microbiota Diversity and Community Structures

**Figure 5:**
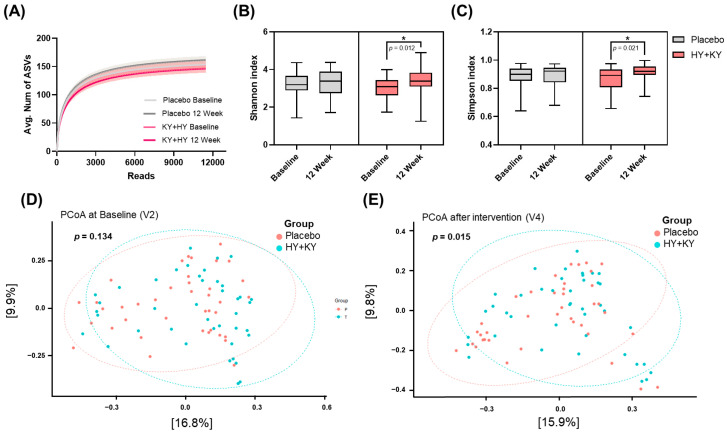
Results of gut microbiota diversity and community structure between the placebo and HY+KY groups. (**A**) Rarefaction curves were represented to evaluate sequencing depth and to confirm sampling saturation across the samples. Box plots represent changes in alpha diversity before and after the 12-week intervention, as measured by the (**B**) Shannon index and (**C**) Simpson index. Data are presented as mean ± SD. *p*-values were analyzed using the Wilcoxon signed-rank test. * *p* < 0.05. Principal coordinates analysis (PCoA) scatter plots illustrate the beta diversity of the placebo and HY+KY groups at (**D**) baseline and (**E**) after 12 weeks.

To investigate the impact of the 12-week probiotic intervention on the gut microbiome, we analyzed the microbial diversity and community structure at baseline and 12 weeks (Figure 5). The rarefaction curves for all samples reached a plateau, indicating that sequencing depth was sufficient to capture the majority of the microbial species in each group (Figure 5A). Microbiome analysis revealed that the HY+KY intervention significantly increased alpha diversity, evidenced by elevated Shannon (*p* = 0.012) and Simpson (*p* = 0.021) indices (Figure 5B,C). Beta diversity was evaluated via PCoA (Figure 5D,E). At baseline, no significant difference in microbial community structure was observed between the HY+KY and placebo groups (*p* = 0.134). Following the 12-week intervention, however, the gut microbiome composition of the two groups became significantly distinct (*p* = 0.015).

### 3.6. Changes in Gut Microbial Composition at the Family and Genus Levels

**Figure 6:**
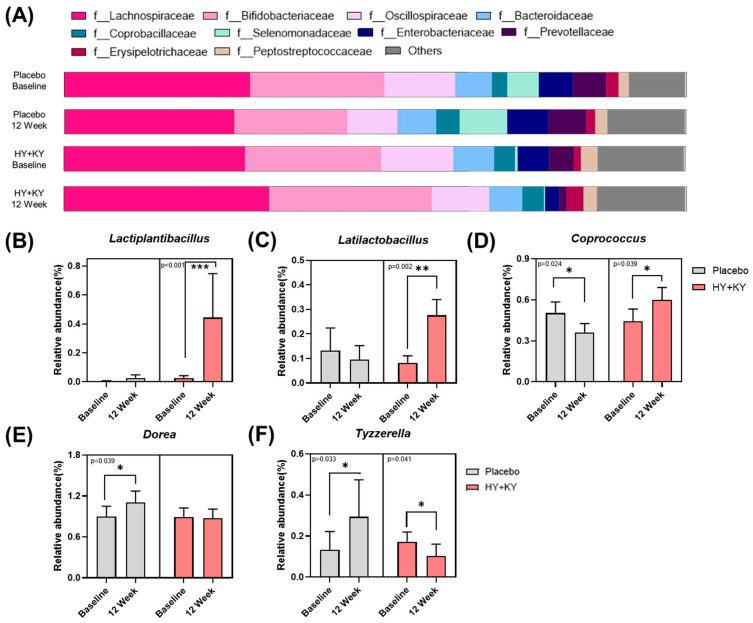
Taxonomy composition of the gut microbiota at the family and genus levels. (**A**) Relative abundance (%) of top 10 bacterial families at baseline and after 12-week of intervention. (**B**–**F**) Comparison of relative abundance (%) of 5 fecal microbiota genera at baseline and 12-week in each group. (**B**) *Lactiplantibacillus*. (**C**) *Latilactobacillus*. (**D**) *Coprococcus*. (**E**) *Dorea*. (**F**) *Tyzzerella*. Genera are shown in the bar plot as mean ± SD. The *p*-values for within-group differences between baseline and 12 weeks were analyzed using the Wilcoxon signed-rank test. * *p* < 0.05, ** *p* < 0.01, *** *p* < 0.001.

The taxonomic composition of the gut microbiota was further analyzed to identify specific shifts at the family and genus levels after the 12-week intervention (Figure 6). To evaluate the changes in the gut microbiome composition, the relative abundance of the 10 most abundant families with the highest mean relative abundance across all samples were analyzed (Figure 6A). In the placebo group, the relative abundance of *Lachnospiraceae* shifted from 29.87% at baseline to 27.32% at 12 weeks and *Bifidobacteriaceae* decreased from 21.59% to 18.20%. However, *Enterobacteriaceae* increased from 5.52% to 6.59% during the same period. In the HY+KY group, distinct patterns were observed. The relative abundance of *Lachnospiraceae* increased from 29.17% at baseline to 32.99% at 12 weeks, and *Bifidobacteriaceae* increased from 21.85% to 26.15%. Notably, the abundance of *Enterobacteriaceae* in the HY+KY group was reduced by more than half, falling from 5.11% to 2.33%.

Analysis of the genus level composition revealed several significant within-group changes (Figure 6B–F). The relative abundance of *Lactiplantibacillus* significantly increased in the HY+KY group (*p* < 0.001). Similarly, *Latilactobacillus* showed a significant increase in the HY+KY group after the 12-week intervention (*p* = 0.002). For the *Coprococcus*, a significant increase was observed in the HY+KY group (*p* = 0.039), whereas it significantly decreased in the placebo group (*p* = 0.024). The relative abundance of *Dorea* significantly increased in the placebo group (*p* = 0.039), but no significant variation was found in the HY+KY group. Finally, *Tyzzerella* exhibited contrasting patterns between the two groups, with a significant increase in the placebo group (*p* = 0.033) and a significant reduction in the HY+KY group (*p* = 0.041).

### 3.7. Association of Gut Microbiota with Lipid Metabolism Improvements

**Figure 7:**
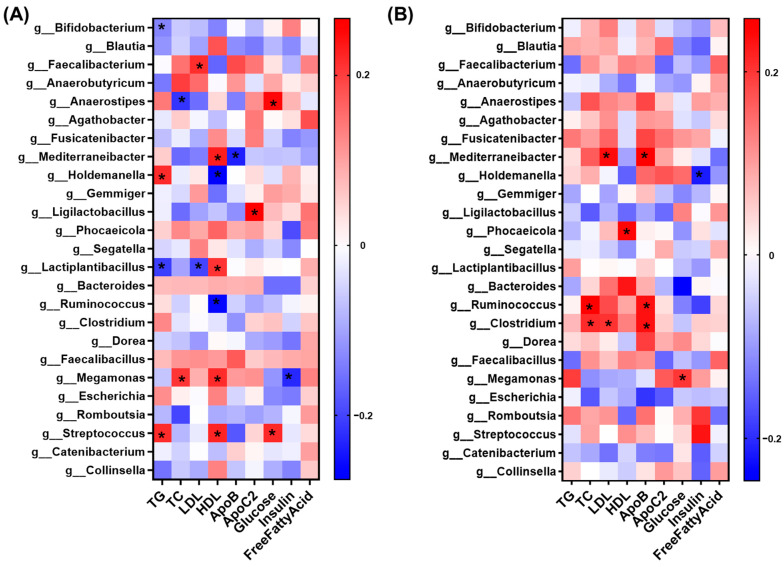
Heatmap graphs between gut microbiota and clinical parameters. Heatmaps illustrate the correlations between the relative abundance of gut microbial genera and clinical biomarkers in the (**A**) HY+KY group and (**B**) placebo group after the 12-week intervention. The color intensity represents the Spearman correlation coefficient, where red indicates a positive correlation and blue indicates a negative correlation. Heatmaps showing the abundance of the top 25 differential genera. * *p* < 0.05.

To evaluate the relationship between the altered gut microbiota and the improvement in metabolic parameters, Spearman correlation analysis was performed at the genus level (Figure 7). In the HY+KY group, *Bifidobacterium* and *Lactiplantibacillus* exhibited significant negative correlations with TG levels, while *Holdemanella* and *Streptococcus* also showed significant associations (Figure 7A). LDL was positively correlated with *Lactiplantibacillus* and negatively correlated with *Faecalibacterium*. Other lipid metabolism markers were significantly associated with a diverse and evenly distributed range of microorganisms.

In contrast, TG in the placebo group did not show any significant correlation with gut microbiota (Figure 7B). LDL in the placebo group was positively correlated with *Mediterraneanibacter* and *Clostridium*, and overall, only a limited number of genera exhibited significant associations with lipid metabolism markers. Notably, in the HY+KY group, 10 out of the top 25 genera accounted for a total of 19 significant correlations, whereas the placebo group showed 10 significant correlations involving six genera.

### 3.8. Network Analysis Between Microbiome and Lipid Parameters

**Figure 8:**
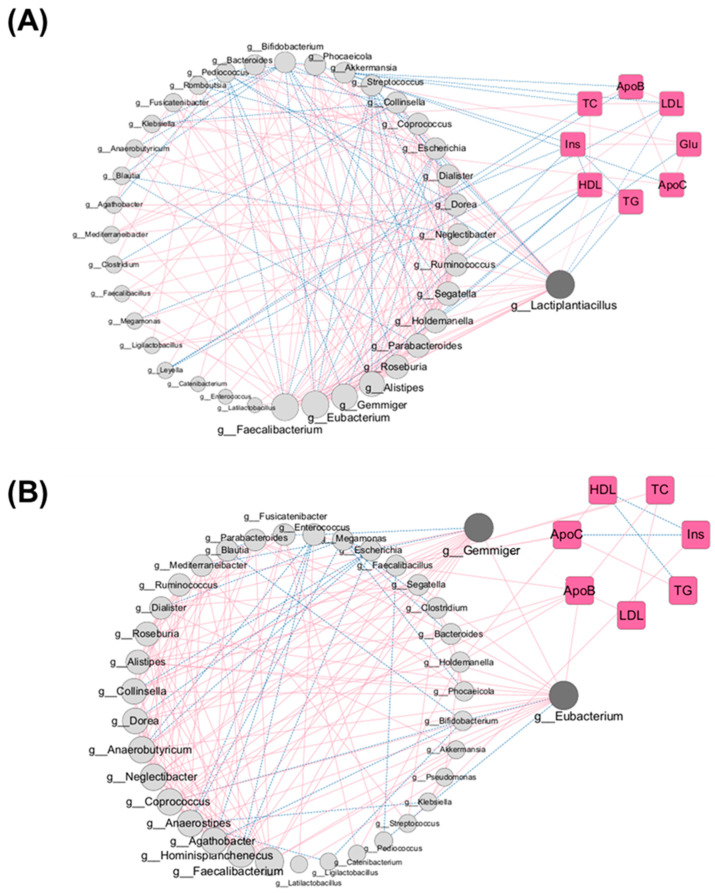
Network analysis between gut microbiota and clinical parameters. Network plots illustrate the significant correlation between gut microbial genera (gray circles) and clinical biomarkers (pink squares) in the (**A**) HY+KY group and (**B**) placebo group. The size of the microbial nodes represents their edge counts, while the lines (edges) indicate significant Spearman correlations (*p* < 0.05). Solid red lines represent positive correlations, and dashed blue lines represent negative correlations, with node sizes scaled proportionally to the interaction count (Total degree) of each genus.

Network analysis was performed to visualize the structural interactions between the gut microbiota and lipid metabolism (Figure 8). The networks for both the HY+KY and placebo groups were composed of two functional modules representing the microbiome and clinical lipid biomarkers, with microorganisms exhibiting the highest number of inter-modular interactions identified as central hubs. In the HY+KY group, *Lactiplantibacillus* was identified as the central node, while *Gemmiger* and *Eubacterium* served as hubs in the placebo group. The topological properties of these networks are summarized in Table 3.

Compared to the placebo group, the HY+KY group exhibited a more complex and integrated network architecture, characterized by a higher number of edges (209), network density (0.106), and clustering coefficient (0.24). Furthermore, centrality analysis demonstrated that *Lactiplantibacillus* possessed a higher degree of correlation and significantly greater betweenness centrality (0.401) and closeness centrality (0.889) compared to the placebo hubs, confirming its superior role as a bridge between the two modules.

Regarding specific associations, *Lactiplantibacillus* exhibited significant negative correlations with TG and LDL, and a positive correlation with HDL. In contrast, *Gemmiger* showed no significant interactions with clinical parameters, and *Eubacterium* was only negatively associated with ApoB.

## 4. Discussion

The present study was conducted to evaluate the efficacy of a 12-week supplementation with a probiotic mixture of *L. curvatus* HY7601 and *L. plantarum* KY1032 on fasting serum TG levels and to elucidate the correlations between gut microbiota modulation and improved lipid profiles in mild HTG. Subjects consuming HY+KY for 12 weeks exhibited a significant reduction in fasting serum TG and LDL levels, accompanied by a concurrent increase in ApoC2 concentrations. In addition, a reduction in fasting Glu levels contributed to the comprehensive improvement of the systemic lipid and metabolic profiles. Furthermore, HY+KY enhanced gut microbial diversity and induced a distinct reconfiguration of the community structure; notably, co-occurrence network analysis identified *Lactiplantibacillus* as a central node, which effectively mediated the favorable modulation of lipid-related biomarkers.

Beyond its established anti-obesity effects [32,37,38], the metabolic benefits of HY+KY have been extensively elucidated through various in vitro and in vivo models [12,39]. Previous in vivo research demonstrated that the HY+KY complex effectively modulates energy metabolism in adipose tissue and facilitates cholesterol disposal [12]. Clinically, supplementation in hyper-triglyceridemic patients has been shown to significantly lower serum TG levels while elevating apolipoprotein A-V (ApoAV)—a critical regulator of TG-rich lipoprotein clearance [32]. While previous clinical investigations have primarily focused on diagnosed patient cohorts, the current study provides robust evidence for the efficacy of HY+KY even in individuals with TG levels below the pathological threshold (<200 mg/dL) [40]. By improving TG concentrations and associated metabolic biomarkers in this borderline population, our findings suggest that HY+KY can function as a proactive intervention to thwart the progression toward overt HTG. Furthermore, the consistent clinical reproducibility of these lipid-lowering effects reinforces the metabolic functionality of the HY+KY probiotic complex, substantiating its efficacy as a specific nutritional strategy for systemic lipid management.

Our findings demonstrate a progressive and significant reduction in both fasting TG and LDL levels throughout the 12-week intervention period, reinforcing the lipid-lowering potential of the HY+KY complex. Notably, the TG-lowering effect was initiated as early as week 6 and was sustained until the end of the trial, while a significant improvement in LDL concentrations was concurrently achieved by week 12. In a prior investigation by Fuentes et al. (2013), *L. plantarum* supplementation resulted in a significant reduction in TG levels at week 6, which aligns with our early-onset findings; however, this significance was not sustained through week 12, and LDL levels only showed a significant decrease at the end of the trial [41]. Similarly, a clinical study on *L. plantarum* K50 by Sohn et al. reported a significant reduction in TG levels at week 12, yet this result was confounded by a simultaneous significant increase in the placebo group, and no meaningful changes were observed in LDL concentrations [42]. Elevated TG levels are closely linked to the formation of small, dense LDL (sdLDL), which are more prone to oxidation and possess a higher potential for arterial wall penetration than larger LDL particles [43]. Therefore, the simultaneous reduction in both TG and LDL is critical for comprehensively mitigating the atherogenic burden and significantly lowering the overall risk of cardiovascular disease [44]. Consequently, the concurrent improvement of TG and LDL observed in our study demonstrates that HY+KY supplementation contributes to the stabilization of the lipid profile and the promotion of metabolic health by effectively reducing the total pool of pro-atherogenic lipoproteins.

The observed improvements in the systemic lipid profile are further substantiated by favorable modulations in ApoC2 and fasting glucose levels. In this study, HY+KY supplementation led to a significant elevation in ApoC2 alongside a concurrent reduction in fasting glucose by week 12. ApoC2 serves as an essential cofactor for lipoprotein lipase (LPL), the primary enzyme that facilitates the hydrolysis and subsequent clearance of TG-rich lipoproteins from the systemic circulation [45]. Furthermore, the reduction in fasting Glu concentrations reflects an enhancement in systemic insulin sensitivity, a process known to effectively suppress the hepatic overproduction of very low density lipoprotein (VLDL)-TG particles [46]. Interestingly, serum insulin levels remained stable without significant fluctuations throughout the study period. This phenomenon likely indicates that the HY+KY complex improves Glu disposal by enhancing insulin receptor sensitivity or through non-insulin-dependent pathways—such as the modulation of glucagon-like peptide-1 (GLP-1) secretion or the production of short-chain fatty acids (SCFAs)—rather than by stimulating additional insulin secretion [47]. Collectively, the coordinated association of these enzymatic and metabolic markers suggests that HY+KY intervention promotes a systematic restoration of lipid homeostasis rather than a transient reduction in TG levels. When evaluating the robustness of the intervention via FAS analysis, the primary efficacy on TG remained clear. However, the significance for LDL and glucose was less pronounced compared to the PPS results. In particular, glucose levels remained relatively stable, which is expected given that the baseline levels of the participants were within the normal range. The lack of significance for these markers in the FAS likely stems from the inclusion of subjects who underwent significant lifestyle changes or medication use during the study (such as statin) [7], which are known to confound metabolic parameters. Nevertheless, the consistent direction of change across both PPS and FAS populations reinforces the overall metabolic benefits of HY+KY.

The systemic restoration of lipid homeostasis observed in our study is fundamentally rooted in the adaptive remodeling of the intestinal ecosystem [48,49]. Recent paradigms in metabolic research have increasingly shifted toward the integrated analysis of gut microbiome dynamics alongside clinical lipidomics to provide an overall view of how probiotic interventions modulate host health [10]. This integrative approach is now considered essential for understanding the complex relationship between the gut microbiota composition and systemic lipid metabolism [50]. To elucidate this inter-systemic connection, we examined the structural variations in the gut microbiota following the intervention (Figure 5). Our findings revealed that 12 weeks of HY+KY supplementation led to a significant increase in α-diversity compared to baseline, indicating an enhancement in microbial community richness and stability. Furthermore, while no significant differences in β-diversity were observed between the HY+KY and placebo groups at baseline (*p* = 0.134), a distinct and significant separation in the distribution of the microbial community emerged after the 12-week period (*p* = 0.015, PCoA). These results are in close agreement with previous clinical study demonstrating that 12-week supplementation with the HY+KY effectively shifts the intestinal landscape toward a more diverse and stable state [51]. Enhanced gut microbial diversity is typically associated with increased metabolic flexibility and a reduced risk of lipid disorders precipitated by intestinal dysbiosis [52,53]. Moreover, these findings highlight the potent capacity of HY+KY to fundamentally restructure the gut microbial community, establishing a microbial environment that is more favorable for systemic lipid management and metabolic homeostasis [50].

To identify the specific microbial taxa driving the observed shifts in community structure, we compared the relative abundance of the top 10 bacterial families (Figure 6A). The most prominent taxonomic shifts occurred in the families *Lachnospiraceae* and *Bifidobacteriaceae*. Specifically, while their relative abundances decreased in the placebo group over the 12-week period, they were significantly enriched in the HY+KY group. Conversely, the *Enterobacteriaceae* and *Prevotellaceae* families exhibited an upward trend in the placebo group but were effectively suppressed in the HY+KY group. *Lachnospiraceae* and *Bifidobacteriaceae* are well-established beneficial taxa that play pivotal roles in promoting metabolic health. Members of the *Lachnospiraceae* family are primary producers of SCFAs, particularly butyrate, which is known to strengthen the intestinal barrier and exert potent anti-inflammatory effects [54]. Similarly, *Bifidobacteriaceae* contribute significantly to gut homeostasis and have been clinically shown to improve lipid profiles and enhance insulin sensitivity [55]. In contrast, the expansion of *Enterobacteriaceae* and *Prevotellaceae* is frequently associated with unfavorable metabolic conditions. An overgrowth of *Enterobacteriaceae* serves as a prominent marker of gut dysbiosis and is closely linked to systemic inflammation and metabolic disorders [56]. Furthermore, certain expansions within the *Prevotellaceae* family have been identified as associated with chronic inflammation and impaired Glu metabolism in various clinical settings [57].

An analysis at the genus level revealed that HY+KY supplementation induced a strategic and beneficial shift in the intestinal landscape (Figure 6B–F). The administered probiotic genera, *Lactiplantibacillus* and *Latilactobacillus,* showed a significant increase in the HY+KY group after 12-week intervention (*p* < 0.001 and *p* = 0.002, respectively) indicating a significant enrichment of these taxa within the gut environment. While 16S rRNA sequencing identifies changes at the genus level, these results suggest that the administered probiotics effectively modulated the microbial composition. These genera are well-documented for their lipid-lowering effects, contributing to the reduction in systemic TG through the modulation of metabolic pathways [34,58]. Furthermore, the beneficial genus *Coprococcus* significantly increased in the HY+KY group (*p* = 0.039) while decreasing in the placebo group (*p* = 0.024). As a key producer of SCFAs, *Coprococcus* is recognized for its essential role in maintaining lipid homeostasis and reducing the risk of metabolic syndrome [59,60]. Conversely, HY+KY intervention effectively suppressed the expansion of opportunistic genera associated with metabolic risk. While *Dorea* and *Tyzzerella* significantly increased in the placebo group (*p* = 0.039 and *p* = 0.033, respectively), *Tyzzerella* significantly reduced in the HY+KY group following the 12-week period (*p* = 0.041). Notably, both *Dorea* and *Tyzzerella* have been identified as microbial markers for high cardiovascular risk and pro-atherogenic dietary patterns, with *Dorea* specifically showing positive correlations with obesity and intestinal inflammation [61,62,63,64]. In summary, the selective enrichment of these beneficial taxa and the concomitant inhibition of metabolic risk markers underscore the efficacy of HY+KY in restoring the gut environment toward a metabolically favorable state.

Spearman correlation analysis, visualized through heatmaps, serves as an integrative tool for deciphering the complex interplay between microbial community shifts and host clinical phenotypes, effectively identifying specific microbial taxa associated with metabolic health [65]. In the present study, Figure 7 was utilized to explore whether the taxonomic shifts within the gut microbiota hold significant clinical implications and to evaluate if the metabolic improvements observed following HY+KY supplementation were indeed statistically associated with these microbial changes. The taxonomic shifts from Figure 6 were further evaluated by the heatmap analysis. *Lactiplantibacillus*, significantly enriched in the HY+KY group, showed potent negative correlations with TG and LDL and positive correlations with HDL, suggesting its potential role in favorable lipid modulation. Conversely, *Dorea,* which expanded in the placebo group, exhibited opposing clinical trends, negatively impacting the lipid profile. These changes substantiate that *Lactiplantibacillus* is a significant microbial genus for lipid improvement, while *Dorea* acts as a genus associated with negative metabolic impacts. Additionally, the well-recognized beneficial genus *Bifidobacterium* exhibited a negative correlation with TG levels, whereas *Streptococcus*, a potential pathogen, was positively associated with these lipid markers [66,67]. In contrast, the placebo group was characterized by *Clostridium*, which showed significant positive correlations with TC, LDL, and ApoB, dominating the group’s unfavorable metabolic state. These observations are consistent with the clinical findings of Zhou et al., who reported that an increased abundance of *Clostridium* sp. is positively correlated with elevated levels of LDL and TC [68]. The robustness of the HY+KY intervention is further evidenced by the higher density of microbial–clinical interactions; the HY+KY group yielded 19 significant correlations across 10 genera, whereas the placebo group showed only 10 correlations across six genera. This quantitative difference suggests that HY+KY supplementation does not merely alter microbial abundance but is associated with a more integrated and metabolically favorable network related to systemic lipid metabolism [69].

To provide a comprehensive perspective extending beyond individual correlations, we performed network analysis to visualize the integrated relationships between gut microbiota and clinical parameters, including both microbe–microbe and microbe–host interactions [31,70]. Figure 8 organizes the microbiome and clinical indicators into distinct functional modules, identifying the genera with the highest number of inter-modular edges as central hub nodes. In the HY+KY group, *Lactiplantibacillus* was identified as the primary hub node; notably, this genus was among the taxa that significantly increased following treatment and, consistent with the results in Figure 7, acted as a key taxon driving improvements in clinical lipid profiles. In contrast, the hubs in the placebo group, *Gemmiger* and *Eubacterium*, demonstrated limited functional relevance; *Gemmiger* showed no significant association with clinical parameters in Figure 7, while *Eubacterium* was uniquely correlated only with ApoB in the network. These results suggest that while the microbiome shifts in the HY+KY group exhibit a highly integrated structure where central nodes correlate closely with favorable clinical outcomes, the shifts in the placebo group likely reflect mere temporal fluctuations in gut microbial composition that lack functional integration with host metabolic improvements [71,72]. This disparity reinforces the role of HY+KY as a potent modulator that effectively synchronizes the gut ecosystem with systemic lipid catabolism.

The global topological properties of the co-occurrence networks provide critical insights into the stability and functional integration of the gut ecosystem [73]. In Table 3, the HY+KY group exhibited a significantly more complex and robust network architecture compared to the placebo group, characterized by a higher number of edges (209 vs. 173), increased network density (0.106 vs. 0.091), and a higher clustering coefficient (0.240 vs. 0.203). In microbial ecology, such structural reinforcement indicates a resilient community that is more efficiently coordinated to perform specific metabolic functions [74]. Crucially, this enhanced architecture in the HY+KY group is anchored by the superior centrality metrics of *Lactiplantibacillus*. With its superior betweenness centrality (0.401) and closeness centrality (0.889), *Lactiplantibacillus* serves as a key topological bridge, reflecting robust statistical links between microbial members and clinical lipid biomarkers. In contrast, while the placebo group also possessed hub nodes such as *Gemmiger* and *Eubacterium*, these taxa failed to establish significant clinical relevance. This disparity suggests that HY+KY supplementation does not merely alter the abundance of specific bacteria but fundamentally remodels the gut environment into a highly synchronized regulatory system. While the placebo network remains fragmented with hubs that reflect mere temporal fluctuations, the HY+KY network utilizes its central node to actively drive favorable clinical outcomes [67,68]. These findings demonstrate that the probiotic intervention effectively optimizes the systemic modulation of lipid metabolism by establishing a stable, hub-driven microbial–clinical network.

Our findings suggest that HY+KY supplementation contributes to the restoration of lipid homeostasis, potentially through the establishment of a stable microbial–clinical network. However, this study has several limitations. In particular, the lack of direct measurement of microbial metabolites, such as short-chain fatty acids (SCFAs), limits our ability to fully elucidate the underlying mechanisms. Another limitation is that habitual consumption of kimchi—the source matrix of the probiotic strains—was not explicitly quantified. Nevertheless, randomized allocation successfully minimized potential confounding, as evidenced by the comparable total caloric intake and statistically equivalent baseline abundances of *Lactiplantibacillus* and *Latilactobacillus* between the groups. Future studies incorporating metabolomic analyses are warranted to better define the biochemical pathways through which HY+KY influences host metabolic health. Taken together, these findings provide a scientific basis for targeting the gut–metabolic axis as a potential strategy for managing dyslipidemia in clinical settings.

## 5. Conclusions

In conclusion, this 12-week, randomized, double-blind, placebo-controlled trial demonstrates that the probiotic complex HY+KY is a safe and effective dietary intervention for individuals with mild HTG. Our findings indicate that daily supplementation significantly stabilizes TG, TC, and Glu levels, while simultaneously enhancing ApoC2-mediated lipid clearance. Importantly, the consistent reduction in fasting TG levels across both the strictly compliant PPS and the FAS underscores the clinical robustness of this intervention. Rather than functioning as a primary treatment for clinical-stage disorders, this intervention shows significant prophylactic potential by maintaining metabolic homeostasis and preventing the progression of borderline imbalances into more severe cardiovascular risks. This systemic stabilization is fundamentally driven by a more integrated and resilient gut microbiota network, modulated by the central node *Lactiplantibacillus*. Collectively, these results support the use of the HY+KY complex as a promising functional food strategy for the early prevention and stable management of metabolic parameters, offering a sustainable approach to long-term cardiovascular and metabolic health in the general population.

## Figures and Tables

**Table 1:** Baseline characteristics of participants.

Variables	Placebo (*n* = 39)	HY+KY (*n* = 41)	*p*-Value
Sex (M/F)	16/23	18/23	0.798
Age (years)	44.72 ± 12.21	45.71 ± 11.52	0.71
Drinker (Yes/No)	20/19	24/17	0.521
Smoker (Yes/No)	8/31	5/36	0.32
Height (cm)	166.37 ± 7.98	166.99 ± 9.78	0.76
Weight (kg)	71.06 ± 9.44	75.64 ± 4.78	0.336
BMI (kg/m^2^)	25.67 ± 3.15	26.29 ± 3.81	0.438
SBP (mmHg)	124.97 ± 10.55	124.46 ± 9.15	0.817
DBP (mmHg)	77.26 ± 10.13	75.73 ± 7.86	0.453
Heart rate (beats/min)	78.74 ± 11.4	76.71 ± 11.58	0.431

Results are means ± SD. A chi-square test was performed on categorical variables. An independent *t*-test was performed on continuous variables.

**Table 2:** Safety assessments between baseline and after 12 weeks.

Variables	Placebo (*n* = 39)		HY+KY (*n* = 41)		*p*-Value	
	Baseline	12 Week	Baseline	12 Week	Baseline ^a^	12 Week ^b^
	Changes		Changes		Changes ^c^	
RBC (10^6^/μL)	4.64 ± 0.38	4.63 ± 0.36	4.68 ± 0.38	4.72 ± 0.44	0.326	0.634
	−0.01 ± 0.21		0.04 ± 0.2		0.294	
WBC (10^3^/μL)	5.96 ± 1.41	5.74 ± 1.42	6.22 ± 1.73	6 ± 1.31	0.394	0.471
	−0.23 ± 0.97		−0.22 ± 1.35		0.981	
Hemoglobin (g/dL)	14.03 ± 1.23	13.99 ± 1.1	14.29 ± 1.29	14.34 ± 1.41	0.219	0.355
	−0.03 ± 0.62		0.06 ± 0.6		0.512	
Platelet (10^3^/μL)	270.23 ± 51.69	268.21 ± 49.58	248.32 ± 48.91	253.39 ± 46.35	0.171	0.055
	−2.03 ± 31.33		5.07 ± 26.46		0.276	
Neutrophil (%)	54.26 ± 9	55.75 ± 8.39	57 ± 7.8	57.27 ± 7.66	0.399	0.15
	1.48 ± 6.27		0.27 ± 5.58		0.363	
Lymphocyte (%)	35.11 ± 8.83	33.85 ± 7.99	32.75 ± 7.18	32.69 ± 7.26	0.496	0.193
	−1.26 ± 5.63		−0.07 ± 4.44		0.294	
Monocyte (%)	5.4 ± 1.33	5.26 ± 1.39	5.06 ± 1.14	5.13 ± 1.21	0.639	0.223
	−0.14 ± 0.91		0.06 ± 1.12		0.38	
Eosinophil (%)	2.99 ± 1.66	2.94 ± 1.57	3.06 ± 1.54	3 ± 1.72	0.852	0.854
	−0.06 ± 1.02		−0.06 ± 1.48		0.992	
Basophil (%)	0.6 ± 0.3	0.6 ± 0.31	0.59 ± 0.26	0.53 ± 0.25	0.304	0.879
	0 ± 0.22		−0.06 ± 0.24		0.276	
AST (U/L)	25.13 ± 10.14	23.26 ± 10.43	23.15 ± 6.32	22.76 ± 6.26	0.794	0.295
	−1.87 ± 7.52		−0.39 ± 6.28		0.341	
ALT (U/L)	28.23 ± 18.97	27.13 ± 18.49	26.66 ± 16.08	26.39 ± 15.97	0.849	0.69
	−1.1 ± 10.99		−0.27 ± 13.8		0.766	
γ-GTP (U/L)	31.03 ± 30.13	29.77 ± 23.59	30.98 ± 30.56	28.2 ± 17.17	0.733	0.994
	−1.26 ± 9.76		−2.78 ± 25.34		0.726	
BUN (mg/dL)	13.95 ± 3.53	13.44 ± 3.34	14.44 ± 3.24	13.59 ± 2.92	0.832	0.519
	−0.51 ± 3.09		−0.85 ± 2.56		0.592	
Uric acid (mg/dL)	5.65 ± 1.43	5.37 ± 1.13	5.77 ± 1.27	5.69 ± 1.3	0.251	0.682
	−0.28 ± 0.72		−0.09 ± 0.92		0.305	
hs-CRP (mg/L)	1.14 ± 1.94	1.08 ± 2.08	1.31 ± 1.89	1.03 ± 1.41	0.884	0.695
	−0.06 ± 2.6		−0.28 ± 1.63		0.64	
HbA1c (%)	5.67 ± 0.32	5.66 ± 0.31	5.6 ± 0.29	5.61 ± 0.29	0.488	0.37
	−0.01 ± 0.1		0 ± 0.1		0.512	

Changes in hematological and biochemical parameters from baseline to week 12 in the placebo and HY+KY groups. Values are presented as mean ± SD. Baseline values were compared between groups using an independent *t*-test. Within-group changes from baseline to week 12 were analyzed using a paired *t*-test, and between-group differences in changes were assessed using an independent *t*-test. Categorical variables were analyzed using the chi-square test. RBC, red blood cell; WBC, white blood cell; AST, aspartate aminotransferase; ALT, alanine aminotransferase; γ-GTP, gamma-glutamyl transferase; BUN, blood urea nitrogen; hs-CRP, high-sensitivity C-reactive protein; HbA1c, glycated hemoglobin. ^a^: *p*-values indicate between-group comparisons at baseline, ^b^: *p*-values indicate between-group comparisons after the intervention period, ^c^: *p*-values indicate between-group comparison of changes from baseline to 12 weeks.

**Table 3:** Topological properties and centrality metrics of the microbial-metabolic networks.

Category	Variables	HY+KY	Placebo	
Topological properties	Number of nodes	45	44	
	Number of edges	209	173	
	Average number of neighbors	9.289	7.864	
	Network density	0.106	0.091	
	Characteristic path length	2.13	1.947	
	Clustering coefficient	0.24	0.203	
Centrality metrics of hub nodes		*Lactiplantibacillus*	*Gemmiger*	*Eubacterium*
	Degree	22	17	17
	Betweenness Centrality	0.401	0.317	0.389
	Closeness Centrality	0.889	0.773	0.875

Topological parameters were calculated using the Analyzer tool in Cytoscape. Hub genera were identified based on the highest degree and centrality values in each group.

## Data Availability

The datasets generated and analyzed during the current study are available from the corresponding author on reasonable request. The data are not publicly available due to privacy and ethical restrictions.

## Supplementary Materials

The following supporting information can be downloaded at: https://www.mdpi.com/article/10.3390/nu18111713/s1, Table S1: Changes in serum lipid profiles before and after 12-week intervention; Table S2: Changes in apolipoproteins and metabolic biomarkers before and after 12-week intervention; Table S3: Changes in serum lipid profiles, apolipoprotein and metabolic biomarkers before and after 12-week intervention on Full analysis set (FAS).
