# Supplementary material for: Effect of Latilactobacillus curvatus HY7601 and Lactiplantibacillus plantarum KY1032 on Serum Triglyceride Levels and the Gut–Metabolic Axis: A Randomized, Double-Blind, Placebo-Controlled Clinical Trial
Source: Nutrients. 2026 May 27;18(11):1713. doi: 10.3390/nu18111713 (PMC13259388; doi:10.3390/nu18111713)
Supplement: Supplementary file 1 [file nutrients-18-01713-s001.zip › nutrients-4325821-supplementary.pdf]

Table S1. Changes in serum lipid profiles before and after 12-week intervention.

Variables	Placebo (n=39)			95% CI [f]	HY+KY (n=41)			95% CI [f]	*p-value*
	Baseline	6 Week	12 Week		Baseline	6 Week	12 Week		
		Median (Q1-Q3)				Median (Q1-Q3)			
		Changes				Changes			
		p-value				*p-value*			
Triglyceride (mg/dL)	160.28 ± 22.18	171.77 ± 73.29	159.08 ± 79.41	-26.6/24.2	158.61 ± 23.17	144.22 ± 88.68	139.54 ± 54.31	-37.5/-0.6	0.021 [e]
	162 (140-180)	149 (115-228)	145 (105-174)		153 (136-182)	121 (91-170)	132 (103-177)		
		11.49 ± 71.12 [a]	-1.21 ± 78.38 [b]			-14.39 ± 84.27 [a]	-19.07 ± 58.46 [b]		
		0.589 [c]	0.081 [d]			0.002 [c]	0.009 [d]		
Total Cholesterol (mg/dL)	214.74 ± 31.02	217.51 ± 35.18	213.82 ± 36.89	-10.3/8.4	217.39 ± 31.48	213.17 ± 29.75	216.37 ± 29.4	-7.8/5.8	0.985 [e]
	217 (192-237)	218 (192-248)	216 (194-232)		221 (199-231)	211 (199-233)	219 (199-235)		
		2.77 ± 31.08 [a]	-0.92 ± 28.95 [b]			-4.22 ± 20.64 [a]	-1.02 ± 21.61 [b]		
		0.795 [c]	0.851 [d]			0.556 [c]	0.864 [d]		
LDL-Cholesterol (mg/dL)	132.64 ± 24.73	132 ± 31.3	128.41 ± 32.66	-13.0/4.5	129.22 ± 28.45	116.20 ± 23.08	111.34 ± 21.03	-29.0/-6.7	0.023 [e]
	129 (116-144.5)	132 (113-153)	125 (111-151)		125 (112-144)	120 (101-132)	113 (99-128)		
		-0.64 ± 24.92 [a]	-4.23 ± 27.05 [b]			-13.02 ± 24.78 [a]	-17.88 ± 35.35 [b]		
		0.843 [c]	0.669 [d]			0.054 [c]	0.005 [d]		
HDL-Cholesterol (mg/dL)	56.1 ± 13.45	55.41 ± 11.85	55.67 ± 11.69	-2.8/1.9	57.73 ± 12.39	57.78 ± 13.47	57.05 ± 12.86	-2.7/1.3	0.8937 [e]
	54 (47-60)	53 (47-62)	54 (47-62)		58 (46-69)	56 (46-69)	56 (46-67)		
		-0.69 ± 7.78 [a]	-0.44 ± 7.28 [b]			0.05 ± 7.22 [a]	-0.68 ± 6.37 [b]		
		0.883 [c]	0.862 [d]			0.908 [c]	0.717 [d]		

Changes in serum lipid profiles from baseline to 6 weeks and 12 weeks in the placebo and HY+KY groups. Values are presented as mean ± SD and median (interquartile range; Q1–Q3). a: Changes represent the difference from baseline to 6 weeks, b: Changes represent the difference from baseline to 12 weeks, c: *p-value*s (baseline to 6 weeks) by paired t-test or Wilcoxon signed-rank test, d: *p-value*s (baseline to 12 weeks) by paired t-test or Wilcoxon signed-rank test, e: *p-values* for interaction were determined by a LMM evaluating the treatment-by-time interaction, adjusted for baseline participant characteristics, f: 95% CI represents the 95% confidence interval for the 12-week change.

Table S2. Changes in apolipoproteins and metabolic biomarkers before and after 12-week intervention.

Variables	Placebo (n=39)		95% CI [d]	HY+KY (n=41)		95% CI [d]	*p-value*
	Baseline	12 Week		Baseline	12 Week		
	Median (Q1-Q3)			Median (Q1-Q3)			
	Change			Change			
	p-value			*p-value*			
Apolipoprotein B (mg/dL)	105 ± 16.81	105.77 ± 24.15	-5.5/7.0	105.63 ± 17.59	97.2 ± 14.28	-13.9/-2.9	0.496[c]
	104 (95-115)	107 (93.5-120.5)		104 (97-111)	101 (88-108)		
	0.77 ± 19.35 [a]			-8.44 ± 17.48 [a]			
	0.624 [b]			0.129 [b]			
Apolipoprotein CII (mg/dL)	5.53 ± 1.54	5.69 ± 2.01	-0.4/0.7	5.28 ± 1.31	6.04 ± 1.42	0.2/1.3	0.016 [c]
	5.6 (4.8-6.7)	5.5 (4.3-6.9)		5.1 (4.2-6.3)	5.9 (5.2-6.8)		
	0.16 ± 1.89 [a]			0.76 ± 1.66 [a]			
	0.952 [b]			0.034 [b]			
Glucose (mg/dL)	97.08 ± 7.19	98.51 ± 9.2	-1.0/3.8	94.51 ± 7.92	90.27 ± 5.96	-7.2/1.2	0.013 [c]
	96 (92-102.5)	97 (92-102.5)		93 (89-100)	89 (97-93)		
	1.44 ± 7.54 [a]			-4.24 ± 9.54 [a]			
	0.663 [b]			0.021 [b]			
Insulin (μU/mL)	6.57 ± 4.68	7.78 ± 7.56	-1.2/3.6	7.4 ± 6.68	8.26 ± 9.23	-1.5/3.3	0.098 [c]
	5.04 (4.38-8.04)	5.21 (4.12-8.62)		4.73 (3.96-8.56)	5.52 (3.99-8.33)		
	1.21 ± 7.62 [a]			0.85 ± 7.71 [a]			
	0.984 [b]			0.66 [b]			
Free Fatty Acid (μEq/L)	608.36 ± 191.79	573.67 ± 210.19	-111.8/42.4	581.37 ± 228.56	628.81 ± 242.68	-47.1/141.9	0.065 [c]
	565 (509.5-672)	573 (409-735.5)		635 (365-791)	649 (423-787)		
	-34.69 ± 238.03 [a]			47.44 ± 299.41 [a]			
	0.576 [b]			0.371 [b]			

Changes in apolipoproteins and metabolic biomarkers from baseline to 12 weeks in the placebo and HY+KY groups. Values are presented as mean ± SD and median (interquartile range; Q1–Q3). a: Changes represent the difference from baseline to 12 weeks, b: *p-value*s (baseline to 12 weeks) by paired t-test or Wilcoxon signed-rank test, c: *p-values* for interaction were determined by LMM evaluating the treatment-by-time interaction, adjusted for baseline participant characteristics, d: 95% CI represents the 95% confidence interval for the 12-week change.

Table S3. Changes in serum lipid profiles, apolipoprotein and metabolic biomarkers before and after 12-week intervention on Full analysis set (FAS).

Variables	Placebo (n=48)				HY+KY (n=49)				*p-value*[c]
	Baseline	6 Week	12 Week	95% CI[d]	Baseline	6 Week	12 Week	95% CI[d]	
	Median (Q1-Q3)<br>Changes<br>*p-value*				Median (Q1-Q3)<br>Changes<br>*p-value*				
Triglyceride (mg/dL)	160.67 ± 21.24<br>157 (140-181)	165.29 ± 76.67<br>152 (121-211)<br>4.61 ± 76.34[a]<br>0.26[b]	153.37 ± 76.39<br>142 (110-170)<br>-7.31 ± 76.6[a]<br>0.269[b]	-27.6/24.8	156.83 ± 23.2<br>160 (138-179)	145.29 ± 85.3<br>132 (108-167)<br>-14.58 ± 79.07[a]<br><0.001[b]	145.19 ± 57.92<br>138 (113-159)<br>-12.71 ± 62.69[a]<br>0.022[b]	-26.5/-1.1	0.047[e]
Total Cholesterol (mg/dL)	209.92 ± 36.71<br>199 (175-221)	213.9 ± 39.89<br>199 (178-236)<br>3.98 ± 29.22[a]<br>0.629[b]	209.12 ± 41.37<br>202 (165-221)<br>-0.8 ± 28.12[a]<br>0.969[b]	-7.5/11.4	218.67 ± 29.7<br>210 (181-231)	214.29 ± 27.78<br>215 (189-222)<br>-3.63 ± 20.53[a]<br>0.575[b]	217.85 ± 28.9<br>210 (181-224)<br>-1.67 ± 20.53[a]<br>0.777[b]	-9.8/3.8	0.882[e]
LDL-Cholesterol (mg/dL)	128.12 ± 32.1<br>127 (115-136)	128.12 ± 35.93<br>129 (116-137)<br>0 ± 25.58[a]<br>0.722[b]	124.59 ± 36.63<br>126 (115-140)<br>-3.53 ± 26.7[a]<br>0.779[b]	-7.5/11.5	131.94 ± 28.62<br>128 (118-136)	128.83 ± 25.58<br>126 (120-135)<br>-3.25 ± 22.43[a]<br>0.697[b]	123.29 ± 25.99<br>119 (109-126)<br>-8.01 ± 18[a]<br>0.064[b]	-18.6/-1.7	0.076[e]
HDL-Cholesterol (mg/dL)	56.65 ± 13.75<br>54 (53-59)	56.73 ± 12.43<br>56 (54-58)<br>0.08 ± 7.64[a]<br>0.704[b]	55.9 ± 11.79<br>56 (53-58)<br>-0.76 ± 6.93[a]<br>0.991[b]	-2.9/2.1	57.17 ± 12.2<br>57 (55-59)	57.31 ± 13.51<br>56 (54-60)<br>0.35 ± 7.17[a]<br>0.956[b]	56.52 ± 12.53<br>56 (54-59)<br>-0.75 ± 6.28[a]<br>0.678[b]	-2.7/1.6	0.887[e]
Apolipo-protein B (mg/dL)	101.51 ± 21.63<br>100 (98-103)	-<br>0.2 ± 18.6[a]<br>0.792[b]	101.71 ± 26.34<br>101 (99-103)	-6.7/8.1	102.42 ± 19.48<br>102 (95-106)	-<br>3.33 ± 17.32[a]<br>0.129[b]	106.08 ± 17.9<br>105 (99-113)	-5.2/5.8	0.596[c]
Apolipo-protein CII (mg/dL)	5.85 ± 1.64<br>5.4 (4.7-6.0)	-<br>0.02 ± 1.75[a]<br>0.76[b]	5.55 ± 1.96<br>5.1 (4.9-5.9)	-0.3/0.6	5.2 ± 1.27<br>5.3 (4.3-6.2)	-<br>0.53 ± 1.61[a]<br>0.048[b]	5.2 ± 1.5<br>5.2 (5.0-5.7)	0.2/1.4	0.066[c]
Glucose (mg/dL)	95.22 ± 8.13<br>94 (91-99)	-<br>2.1 ± 7.82[a]	97.33 ± 9.06<br>96 (92-101)	-1.1/4.2	93.46 ± 7.97<br>93 (89-96)	-<br>-1.17 ± 7.19[a]	92.1 ± 7.39<br>91 (88-96)	-6.8/1.8	0.112[c]

		0.43 [b]				0.294 [b]			
Insulin (μU/mL)	6.46 ± 4.54<br>6.04 (5.08-7.94)	-<br>1.04 ± 6.84 [a]<br>0.895 [b]	7.5 ± 7.04<br>7.02 (5.82-8.19)	-1.2/4.9	7.61 ± 6.62<br>7.25 (5.53-8.09)	0.89 ± 7.19 [a]<br>0.570 [b]	8.33 ± 8.8<br>8.1 (6.22-8.91)	-1.5/4.0	0.189 [c]
Free Fatty Acid (μEq/L)	599.65 ± 220.24<br>542 (427-655)	-<br>-32.31 ± 250.26 [a]<br>0.589 [b]	567.35 ± 200.13<br>510 (445-672)	-101.6/55.4	589.73 ± 219.1<br>522 (408-712)	-<br>32.11 ± 289.5 [a]<br>0.557 [b]	622.21 ± 229.85<br>588 (492-768)	-38.1/159.2	0.289 [c]

Changes in serum lipid profiles, apolipoprotein and metabolic biomarkers from baseline to 6 weeks and 12 weeks in the placebo and HY+KY groups. Values are presented as mean ± SD and median (interquartile range; Q1–Q3). a: Changes represent the difference from baseline to 6 weeks or 12 weeks, b: *p-value*s (baseline to 12 weeks) by paired t-test or Wilcoxon signed-rank test, c: *p-values* for interaction were determined by LMM evaluating the treatment-by-time interaction, adjusted for baseline participant characteristics, d: 95% CI represents the 95% confidence interval for the 12-week change.
